# WEE1 inhibition targets cell cycle checkpoints for triple negative breast cancers to overcome cisplatin resistance

**DOI:** 10.1038/srep43517

**Published:** 2017-03-06

**Authors:** Hongping Zheng, Fangyuan Shao, Scots Martin, Xiaoling Xu, Chu-Xia Deng

**Affiliations:** 1 Genetics of Development and Disease Branch, National Institute of Diabetes and Digestive and Kidney Diseases, National Institutes of Health, USA; 2 Faculty of Health Sciences, University of Macau, Macau SAR, China; 3 Division of Pre-Clinical Innovation, National Center for Advancing Translational Sciences (NCATS), National Institutes of Health, USA

## Abstract

Cisplatin is one of the most commonly used therapeutic drugs for cancer therapy, yet prolonged cisplatin treatment frequently results in drug resistance. To enhance therapeutic effect of cisplatin, we conducted a high throughput screening using a kinase library containing 704 kinases against triple negative breast cancer (TNBC) cells. We demonstrated that cisplatin activates ATR, CHK1 and WEE1, which shut down DNA replication and attenuate cisplatin induced-lethality. WEE1 inhibition sensitizes TNBCs and cisplatin resistant cancer cells to cisplatin-induced lethality, because it not only impairs DNA replication checkpoint more profoundly than inhibition of ATR or CHK1, but also defects G2-M cell cycle checkpoint. Finally, we demonstrated that combined cisplatin treatment and WEE1 inhibition synergistically inhibits xenograft cancer growth accompanied by markedly reduced expression of TNBC signature genes. Thus targeting DNA replication and G2-M cell cycle checkpoint simultaneously by cisplatin and WEE1 inhibition is promising for TNBCs treatment, and for overcoming their cisplatin resistance.

Cisplatin shows a high cure effect in the treatment of ovarian and testicular cancers, and is also widely used for the treatment of many other malignancies, including head and neck, bladder, esophageal, and small cell lung cancer^1^,^2^,^3^,^4^,^5^,^6^. Cisplatin kill cancer cells via multiple mechanisms, with the best understood mode in promoting the formation of DNA adducts, resulting in inter- and intra-strand cross-linking, followed by the activation of the DNA damage response, cell cycle arrest and the induction of mitochondrial apoptosis^7^,^8^,^9^,^10^,^11^,^12^. However, the development of resistance to cisplatin treatment remains a major obstacle to its clinical application. In the vast majority of cases, tumor cells response well to cisplatin initially, however drug resistance gradually developed and the recurring tumors not only display characteristics of therapeutic resistant but also are highly aggressive^13^,^14^,^15^. In fact, therapeutic failure and tumor recurrence happen clinically to a large fraction of patients with cisplatin treatment. Therefore, it is important to enhance effectiveness of cisplatin and generate novel strategies to target resistant cells.

Generally it is believed that cisplatin-DNA adducts are mainly responsible for cytotoxicity and cell death because they can cause DNA damage and activate apoptotic pathways^1^,^3^,^7^,^16^,^17^,^18^. Although DNA lesions caused by cisplatin are most cytotoxic in S-phase because of their potent inhibition effects on DNA replication, they also activate G2-M checkpoint, leading to G2 arrest which may provide time for cells to repair DNA damage before moving forward to mitotic phase^3^,^19^. Cells that cannot repair damaged DNA properly during this “arrest” period will undergo apoptosis. Interstrand cross-link (ICL) caused by cisplatin has also been well documented, however, the precise molecular mechanisms of cisplatin on replication in S phase still need to be elucidated, although it has been suggested that cisplatin exposure causes replicative stress^20^,^21^. In particular, it remains unclear to which extent the effect of cisplatin on replication contributes to its cytotoxic activity.

Triple negative breast cancer (TNBC), i.e. estrogen receptor [ER]-negative, progesterone receptor [PR]-negative, and HER2-negative, is the most aggressive type of breast cancer^22^,^23^,^24^. Approximately 90% of TNBCs are classified as basal-like breast cancers and the majority of cancers caused by mutations in the breast cancer associated gene 1 (BRCA1) belong to TNBCs^23^. TNBC is characterized by high histological and nuclear grades, a high propensity for metastasis, and poor prognosis^22^,^23^,^24^. Treatment options for TNBCs are limited as they are usually insensitive to most available hormonal or targeted therapeutic agents because of their triple negative nature. Some clinic trials using mono-treatment with cisplatin and combination with other drugs have been reported on TNBCs, but the therapeutic effect is not optimal due to frequently occurred drug resistance^25^,^26^,^27^,^28^.

We hypothesized that the effect of cisplatin on DNA replication plays a critical role in its cytotoxicity and alternations of regulatory factors of DNA replication may change sensitivity of TNBC and even cisplatin-resistant cells to cisplatin. Base on this hypothesis, we conducted a high-throughput siRNA kinome screen to identify kinases when silenced confer sensitivity to cisplatin in two independently maintained MDA-MB-231 TNBC cell lines. After validated by siRNA experiments, kinase hits were examined using specific small-molecule kinase inhibitors for mechanism of synergism. Our screen indicated that while inhibition of ATR, CHK1 or WEE1 serves as a good strategy to overcome cisplatin resistance, WEE1 inhibition is more effective due to its profound effects both on the DNA replication checkpoint and the G2-M cell cycle checkpoint.

## Results

### RNAi screening identified *ATR, CHK1*, and *WEE1* as top synthetic lethal genes for cisplatin in MDA-MB-231 cells

**Figure 1:**
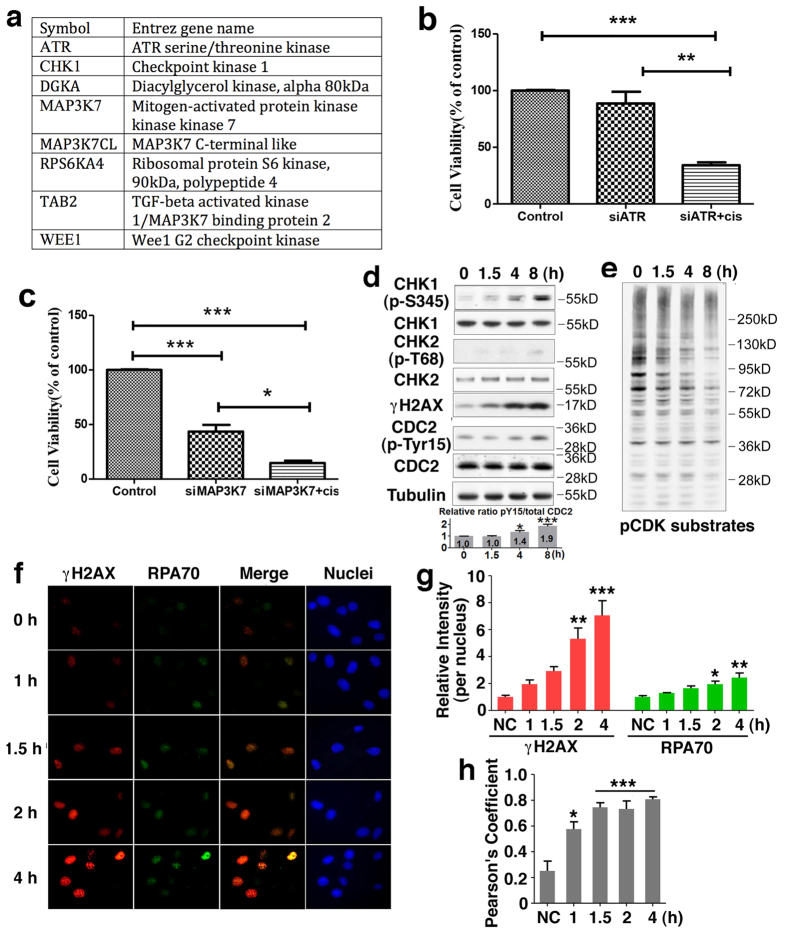
Evaluation of high throughput kinase screens. (**a**) Top 8 candidate kinases that enhance cisplatin cytotoxicity in MDA-MB-231 cells. (**b**,**c**) Follow-up validation using additional siRNAs confirms sensitization with cisplatin mediated by the knockdown of *ATR* (**b**, three siRNA), *MAP3K7* (**c**, two siRNA). (**d**) Cells were treated with cisplatin for indicated time and western blot was done with indicated antibodies. (**e**) pCDK substrate assay conducted at various time points of cisplatin treatment. (**f**) MDA-MB-231 cells were incubated with cisplatin (5 μg/ml) for indicated times and immunostained with the indicated antibodies after pre-extraction. Nuclear DNA was counterstained by DAPI. (**g**) Quantification of average γH2AX or RPA70 values relative to untreated cells of three separate experiments as shown in (**f**) represented as the mean ± SEM. (**h**) Pearson’s coefficient is shown as the quantification of γH2AX and RPA70 co-localization of three separate experiments as shown in (**f**) represented as the mean ± SEM.

To determine which kinases, when silenced, confer sensitivity to MDA-MB-231 cells, we conducted a siRNA screen to identify genes and pathways associated with the cytotoxicity of cisplatin. Screening focused on the human kinome and employed a library targeting 704 human genes with 3 separately arrayed siRNAs per gene in two independently maintained MDA-MB-231 cells. Replicate screens, conducted at separate times, correlated well (Supplementary Fig. 1a) and a comparison of cisplatin treated versus non-treated arms revealed differences suggesting that siRNAs in this screen could modulated cisplatin cytotoxicity (Supplementary Fig. 1b). To select candidate genes that modulate cisplatin activity, the log_2_ ratio of vehicle-treated to cisplatin-treated cell viability was calculated for each siRNA and Redundant siRNA Analysis (RSA) was then used to rank gene candidates in terms of their ability to sensitize MDA-MB-231 to CPT from both cell lines. We identified 8 candidates with p ≤ 0.001 in either replicate screen (Fig. 1a). Additional siRNAs were used to validate the screen result (Fig. 1b,c). Among the top candidates include ATR, CHK1, WEE1 and several other genes, some of which are involved in DNA replication, cell cycle, DNA repair and cancer formation.

Next, we chose to study ATR-CHK1-WEE1 signaling to gain more insight into the impact of cisplatin on cell cycle progression in relation with these well-known cell cycle checkpoint proteins. After cisplatin addition, CHK1 phosphorylation, a target of ATR, gradually increased, while CHK2 phosphorylation, a target of ATM especially in the early stage, showed no obvious change (Fig. 1d). The activation of ATR and CHK1 at some circumstances results in the activation of WEE1 kinase^29^. Consistently, WEE1-mediated CDK1-Y15 phosphorylation, which causes the inactivation of the enzyme, was partially increased (Fig. 1d). Meanwhile overall phospho-CDK substrates levels were decreased (Fig. 1e), indicating that CDK activity was decreased. These changes were correlated with increased γH2AX, a marker for DNA damage in the cisplatin treated cells (Fig. 1d). Thus, this result suggested shortly after cisplatin addition, ssDNA is formed, leading to the ATR activation. To confirm cisplatin treatment indeed triggered the formation of ssDNA and DNA damage, we analyzed cisplatin-treated cells by immunofluorescence staining with an antibody to RPA70, which binds to single strand DNA, and γH2AX. Both RPA70 and γH2AX showed a progressive accumulation upon cisplatin treatment (Fig. 1f). Further quantification using the Pearson’s Correlation Coefficient method^30^ confirmed the strong co-localization of γH2AX and RPA70 signals (Fig. 1g,h). These observations suggest that DNA damage signals were mainly located on ssDNA, which might affect DNA replication.

### Cisplatin treatment leads to replication fork arrested, which can be reversed by inhibition of ATR, CHK1 or WEE1

**Figure 2:**
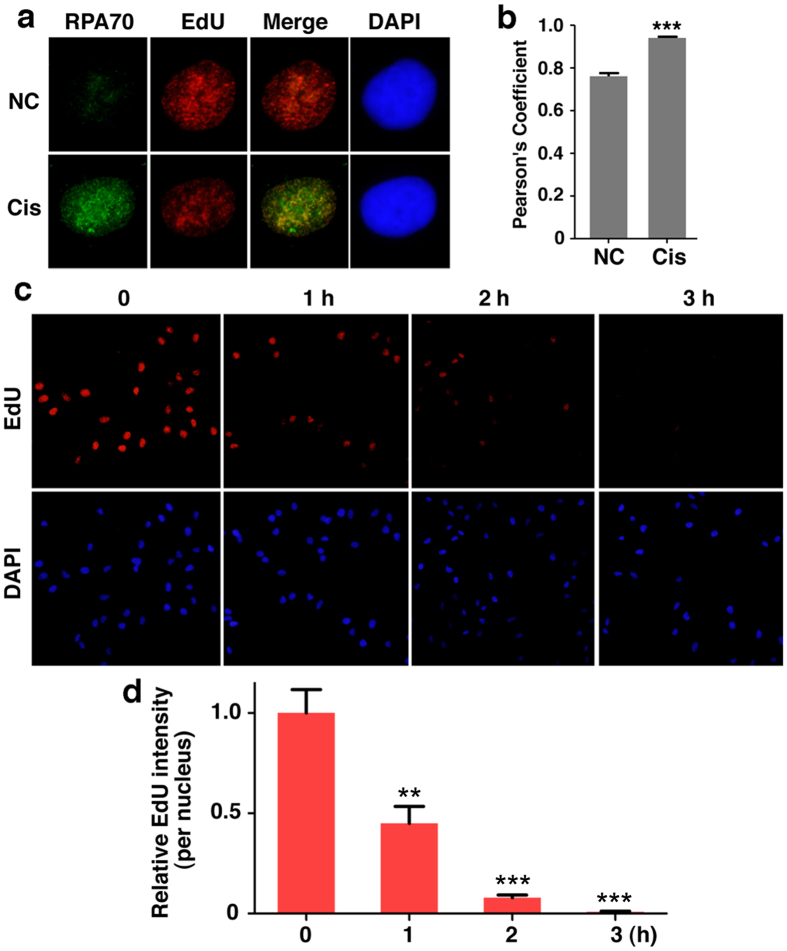
The effect of cisplatin treatment on DNA replication. (**a**) MDA-MB-231 cells were incubated with cisplatin for 30 min or untreated, followed by incubating additionally with EdU for another 20 min. Cells were pre-extracted, and stained for RPA70 by RPA70 antibody, EdU by Click-iT reaction and DNA by DAPI. (**b**) Pearson’s coefficient shown as the quantification of RPA70 and EdU colocalization of three separate experiments as in (**a**) represented as the mean ± SEM. (**c**) MDA-MB-231 cells were treated with cisplatin (5 μg/ml) for indicated times followed by 10 min EdU (10 μM) labeling. Nuclear DNA was counterstained by DAPI and EdU was detected through the Click-iT reaction. (**d**) Quantification of average EdU intensity per nucleus relative to untreated cells of three separate experiments as in (**c**) represented as the mean ± SEM.

To address this further, MDA-MB-231 cells were incubated with cisplatin briefly (30 min) and then combined with nucleoside analog EdU for labeling the replication fork. The data illustrated a strong colocalization of RPA70 and EdU signals (Fig. 2a,b). Thus, the results indicate that cisplatin caused DNA damage was largely confined on replication fork. To examine the possibility that cisplatin impairs DNA replication, cells were followed by a time-course treatment of cisplatin and the data showed that EdU incorporation was dramatically decreased by cisplatin treatment (Fig. 2c,d), suggesting that cisplatin treatment stopped DNA replication.

**Figure 3:**
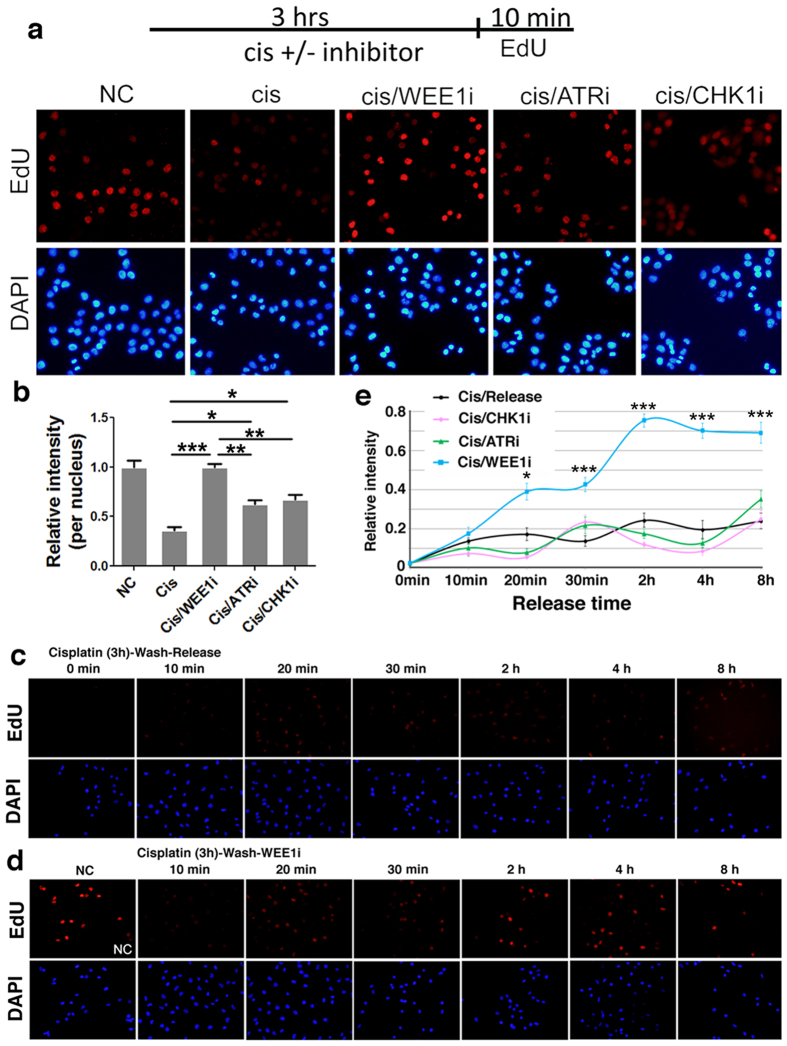
Inhibition of WEE1 activity restores the fork activity more effectively than ATR/CHK1 inhibition. (**a**) MDA-MB-231cells were treated as indicated (top) for 3 hours (EdU was added in the last 10 min of the treatment). Cells were fixed for subsequent staining. Nuclear DNA was counterstained by DAPI. EdU was detected through the Click-iT reaction. (**b**) Quantification of average EdU intensity per nucleus relative to untreated cells of three separate experiments represented as the mean ± SEM. (**c**,**d**) MDA-MB-231 cells were incubated with cisplatin for 3 hours. After washing away cisplatin, the cells were incubated with fresh medium with or without WEE1 inhibitor for indicated times and EdU incorporation for additional 10 min. Cells were fixed for subsequent staining. Nuclear DNA was counterstained by DAPI. EdU was detected through the Click-iT reaction. (**e**) Quantification of average EdU values relative to untreated cells represented as the mean ± SEM. Representative images as shown in (**c**,**d**) and Supplementary Fig. 2.

To confirm if the effects of siRNA against ATR, CHEK1 and WEE1 on cisplatin treated cells are indeed involved in the kinase activity of these proteins, we included specific inhibitors for each of these kinases (ATR inhibitor: ATRi; CHK1 inhibitor: CHK1i; and WEE1 inhibitor: WEE1i) during the course of cisplatin treatment. The data indicated that the addition of these inhibitors markedly reversed the replication impairment caused by cisplatin treatment (Fig. 3a,b). Of note, WEE1i yielded higher intensity of EdU integration in cisplatin treated cells than those caused by ATRi and CHK1i, suggesting that WEE1 has a stronger effect than ATR and CHK1 on DNA replication during cisplatin treatment.

These data indicate that the replicative stress activates these kinases, which immediately halt DNA replication, allowing time for DNA damage repairing. Cell restarts DNA replication once the damages are repaired. To investigate the effect of ATR, CHK1 and WEE1 on DNA replication post cisplatin treatment, we added these inhibitors to cells after cisplatin treatment. The data indicated that cisplatin treatment profound decreased DNA replication during release period of time (Fig. 3c,e). Unexpectedly, we found that replication fork activity was dramatically restored only by WEE1i, but not by ATRi or CHK1i (Fig. 3c–e; Supplementary Fig. 2). This indicates that although all these kinases play important role in restricting DNA replication during cisplatin treatment, WEE1 plays a more crucial role especially during recovery period of time.

### Forced DNA replication causes more DNA damage in cisplatin treated cells

**Figure 4:**
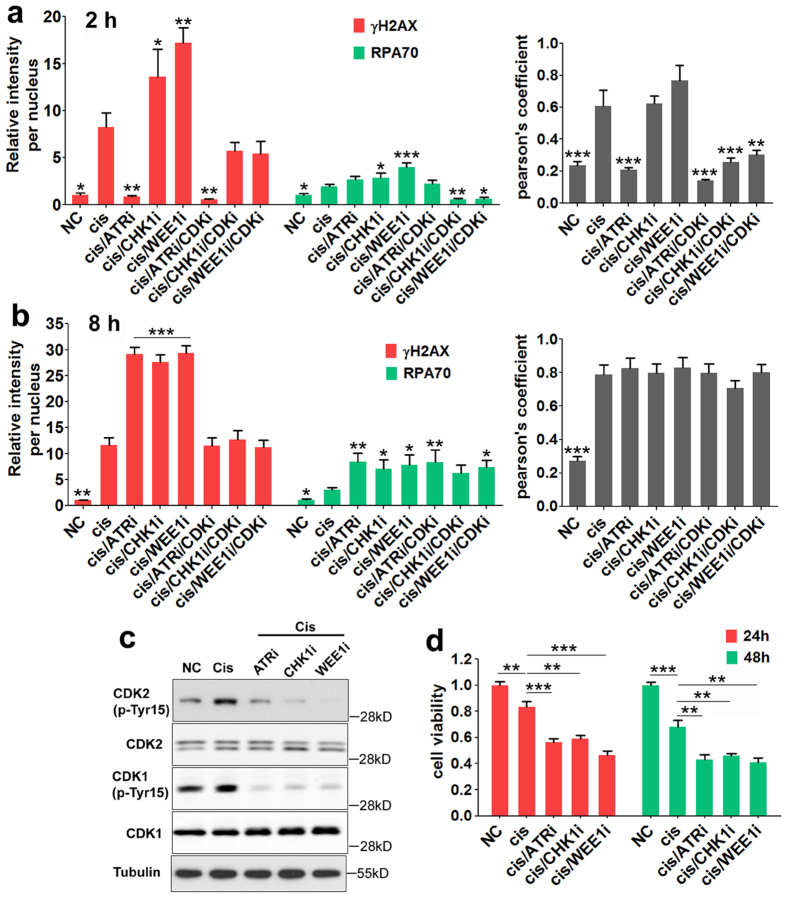
Inhibition of ATR/CHK1/WEE1 activity causes more DNA damage in cisplatin treated cells. (**a**,**b**) Quantification of average γH2AX or RPA70 values relative to untreated cells and Pearson’s coefficient as the quantification of γH2AX and RPA70 co-localization at 2 hours (**a**) and 8 hours (**b**). The data were summarized from three separate experiments and one experiment was shown as in Supplementary Fig. 3. The statistical comparisons were between cisplatin treatment cells and cells of other conditions. (**c**) MDA-MB-231 cells were cultured and treated as in Supplementary Fig. 5, harvested, and subjected to Western blotting with antibodies against CDK1 (phospho-Y15 and total), CDK2 (phospho-Y15 and total), and α-tubulin as loading control. (**d**) MDA-MB-231 cells were cultured and treated as in Supplementary Fig. 5, released for another 24 or 48 hours, and then cells were assessed for cell viability by the MTT assay. Data represent mean ± SEM normalized to cell viability in untreated conditions.

To determine whether the failure of shutting down DNA replication could increase DNA damage when exposed to cisplatin, we treated cells with cisplatin together with each of these inhibitors. We predicted that when applying replication stress by cisplatin treatment, enhanced DNA replication by application of ATRi, CHKi or WEE1i would accelerate ssDNA breakage, which would be marked as DNA damage by γH2AX, and the damage would eventually lead to cell death. To validate this prediction, we measured the signals of RPA70 and γH2AX upon cisplatin treatment in the presence of these inhibitors. After two or eight hour treatment, both RPA70 and γH2AX showed an accumulation upon cisplatin treatment, and CHKi or WEE1i application remarkably enhanced this accumulation (Fig. 4a,b; Supplementary Fig. 3a,b). In the case of ATRi treatment, the lowered instead of increased γH2AX level at 2 h treatment (Fig. 4a) may be contributed to the RPA protection of ssDNA transiently^31^. Yet the level of RPA70 was comparable with those caused by WEE1i and CHK1i (Fig. 4b).

DNA replication occurs at the S phase, to confirm the synergistic action of inhibition of these checkpoint kinase and cisplatin in the S phase, we synchronized cells in the S phase by double thymidine block and analyzed them. Our data indicated that within 4 hours after release, while majority of cells remained in the S phase (Supplementary Fig. 4a), cisplatin treatment activated ATR, CHK1 and WEE1, as evidenced by the increased phosphorylation levels of their immediate targets (Supplementary Fig. 4b), and the phosphorylations were abrogated when the inhibitors of ATR, CHK1 and WEE1 were applied (Fig. 4c). There was no evidence of ATM and CHK2 activation in these cells (Supplementary Fig. 4c). Consistently, but more remarkably, application of ATRi, CHKi or WEE1i significantly increased the γH2AX and RPA70 levels, which implicated the collapse of DNA replication fork and formation of ssDNA (Supplementary Fig. 5a–c). At the same time, the combination of cisplatin with ATRi, CHK1i or WEE1i significantly decreased cell viability compared to cisplatin alone in S-phase cells (Fig. 4d) and increased apoptosis (Supplementary Fig. 5d). Altogether, these data indicate that cisplatin treatment activates ATR, CHK1 and WEE1, which, in turn, shutdown DNA replication as a protection mechanism for enabling cells to repair the damages caused by cisplatin. Inhibition or knockdown of these protection factors forced cells going to DNA replication in the presence of DNA damage, therefore, leading to apoptosis.

### WEE1i exhibits a more effective effect on overcoming cisplatin resistance than ATRi and CHK1i

**Figure 5:**
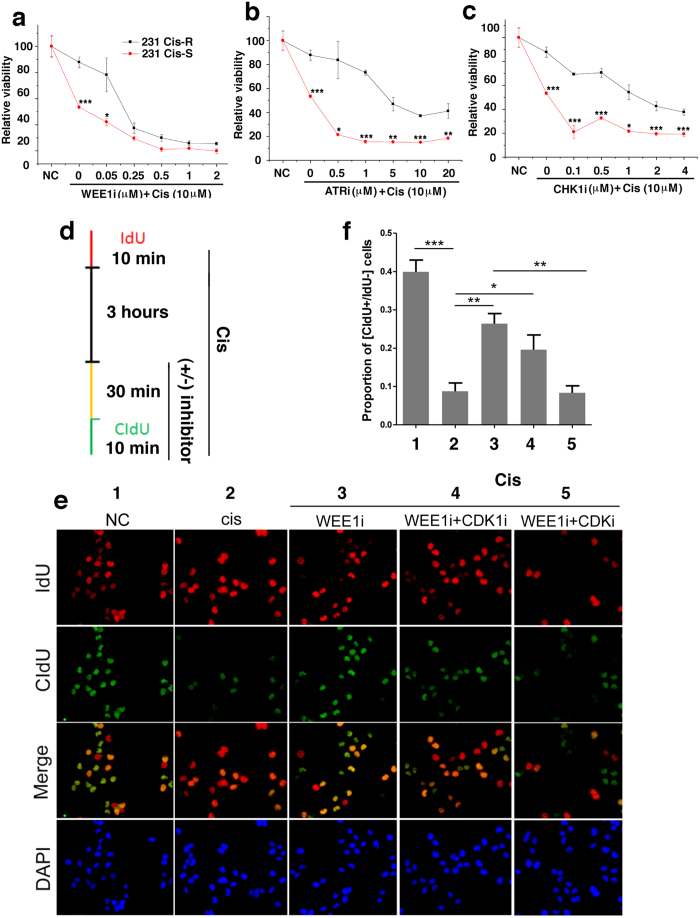
WEE1i exhibits a more effective effect on overcoming cisplatin resistance than ATRi and CHK1i. (**a**–**c**) Relative viability of Cis-R cells and parental MDA-MB-231 (Cis-S) cells in the presence of cisplatin (10 μM) and the increasing concentrations of WEE1i (**a**), ATRi (**b**) and CHK1i (**c**). (**d**) Illustration of the experimental setup, with incorporation of IdU and CldU shown in red and green, respectively. (**e**) As shown in (**d**), MDA-MB-231 cells were incubated with IdU for 10 min as indicated, followed by cisplatin treatment for 3 hours, with or without indicated inhibitor(s) (top) for indicated total 40 min and CIdU was applied for 10 min. Cells were fixed and stained with IdU and CIdU antibodies. Nuclear DNA was counterstained by DAPI. (**f**) Quantification of indicated part of three separate experiments as in (**e**) represented as the mean ± SEM.

Cisplatin resistance is a major obstacle for cancer therapy^13^,^14^,^15^. Next, we sought to determine whether inhibiting activities of these kinases could sensitize cisplatin-resistant cells to the treatment. To this end, we developed several sub-lines of cisplatin-resistant MDA-MB-231 (Cis-R) cells, compared the response of these cells with the parental MDA-MB-231 cells (Cis-S) to cisplatin combined with increased concentration of inhibitors for ATR, CHK1 or WEE1. Our data revealed that increasing concentrations of WEE1i produced increased sensitivities of Cis-R cells to cisplatin; and concentrations of 250 nM and above yielded lethality similar to that of parental cells (Fig. 5a). This effect was more profound than that caused by ATRi and CHK1i (Fig. 5b,c). Consistently, inhibition of WEE1 in Cis-R cells resulted in markedly increased ssDNA coated by RPA70, and DNA damage marked by γH2AX 2 h after the treatment (Supplementary Fig. 6).

To further investigate the impact of WEE1i and cisplatin on DNA replication, we conducted a sequential IdU and CIdU labeling to visualize DNA replication in the presence or absence of cisplatin/WEE1i (Fig. 5d–f). Our result showed that WEE1i was efficient to reverse the effect of cisplatin by triggering CIdU incorporation in the cells, whose DNA replication was shut down by cisplatin (Fig. 5e, compare merged images shown in lanes 1–3). Using a paradigm that the firing of replication origin requires CDKs^32^,^33^, next, we treated cells with several CDK inhibitors. We found that treatment of cells with roscovitine, a pan-CDK inhibitor markedly attenuated the effect of WEE1i (Fig. 5e, lane 5). Because roscovitine could also inhibit G2-M checkpoint in addition to DNA replication we treated cells with inhibitor for CDK1, which is specific for G2-M transition. This yielded a much minor effect on WEE1 inhibitor (Fig. 5e, lane 4). Altogether this data indicates that the reduced DNA replication caused by cisplatin treatment is largely mediated by WEE1 activation in the S-phase but not in the G2-M phase.

**Figure 6:**
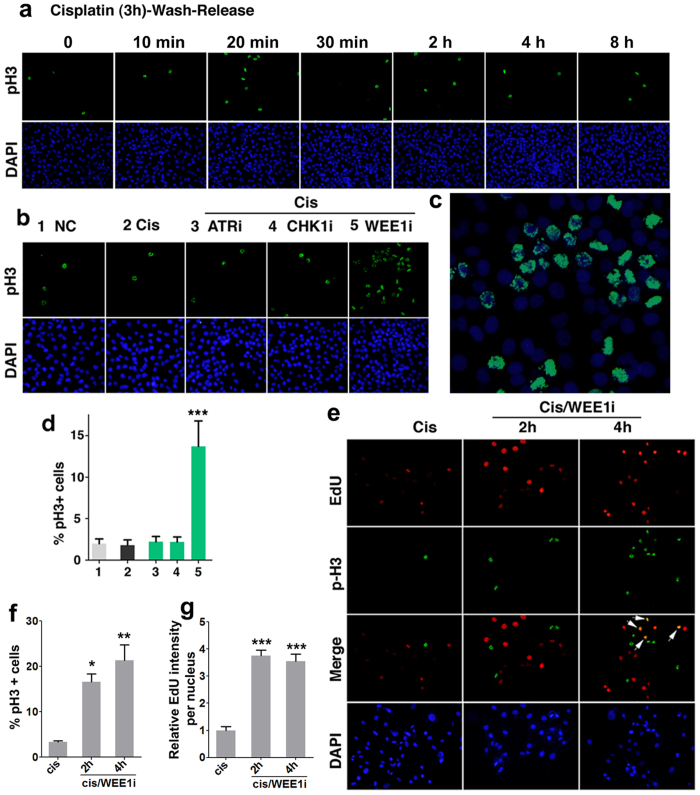
Effect of WEE1i, ATRi, and CHK1i on G2-M cell cycle checkpoint. (**a**) MDA-MB-231 cells were incubated with cisplatin for 3 hours followed by incubation with fresh medium for indicated time. Cells were then fixed and stain with p-H3 antibody. Nuclear DNA was counterstained by DAPI. (**b**) MDA-MB-231 Cells were treated with indicated drug(s) for 4 hours, fixed and stained with DAPI and pH3 antibody. (**c**) Higher power images of cells treated with cisplatin/WEE1i, No. 5). (**d**) Percentages of pH3 positive cells form (**b**) were shown. Data are mean ± SEM from three experiments. (**e**) Cisplatin-resistant 231 Cis-R cells were cultured in medium with cisplatin. The cells were additionally treated with WEE1 inhibitor for indicated times, and EdU was applied in the last 10 min. Then cells were fixed for subsequent staining. Nuclear DNA was counterstained by DAPI, EdU was detected through the Click-iT reaction and pH3 was stained by phospho-Histone 3 antibody. EdU intensity per nucleus were obtained and positive pH3 cells were counted. (**f**) Percentages of pH3 positive cells form (**d**) were shown. Data are mean ± SEM from three experiments. (**g**) Quantification of average EdU values relative to cells incubated only with cisplatin of three separate experiments as in (**d**) represented as the mean ± SEM.

On the other hand, WEE1 is well known for its role regulating G2-M checkpoint through inhibiting CDK1 activity and WEE1 deficiency resulted in premature mitotic entry duo to the activation of CDK1^34^,^35^. Thus, we next investigate the role of WEE1 inhibition in cisplatin treated cells on the mitosis. Our data indicated that cisplatin treatment alone had no obvious effect on M phase entry as no changes of phospho-histone H3 positive (pH3+) cells were observed (Fig. 6a). However, markedly increased mitotic cells were observed upon the treatment WEE1i while such effect was not observed in ATRi or CHK1i treated cells (Fig. 6b–d). Under high power magnification, most pH3+ cells in cis/WEE1i treated population were in the prophase, although some in metaphase and very few in the anaphase (Fig. 6c). We have previously shown that WEE1 deficiency activated CDK1 and the anaphase promoting complex, leading to both premature entry into mitosis and the block in mitosis progression^34^,^35^. The increased population of cells in the mitosis is consistent with these findings.

Because WEE1i enables cisplatin-treated cells underwent DNA replication and also induces premature mitosis entry, next we determined relationship between these two events. Our analysis indicated WEE1 inhibition increased number of EdU+ (S phase) and pH3+ (M-phase) cells compared with mono-treatment of cisplatin (Fig. 6e–g). Of note, 4 hours after WEE1i treatment many cells became EdU/pH3 double positive, indicating they already prematurely entered the mitosis while still in the S phase although such events were not obvious at 2 h (Fig. 6e–g). Altogether, these data indicated that WEE1 plays an essential role in at least two cell cycle checkpoints, i.e DNA replication initiation and G2 to M entry, which underscores the importance of WEE inhibition as a strategy for cancer therapy and accounts for the reason why WEE1i is more potent than ATRi and CHKi.

### Antitumor efficacy of cisplatin and WEE1i in human breast cancers both *in vitro* and *in vivo*

**Figure 7:**
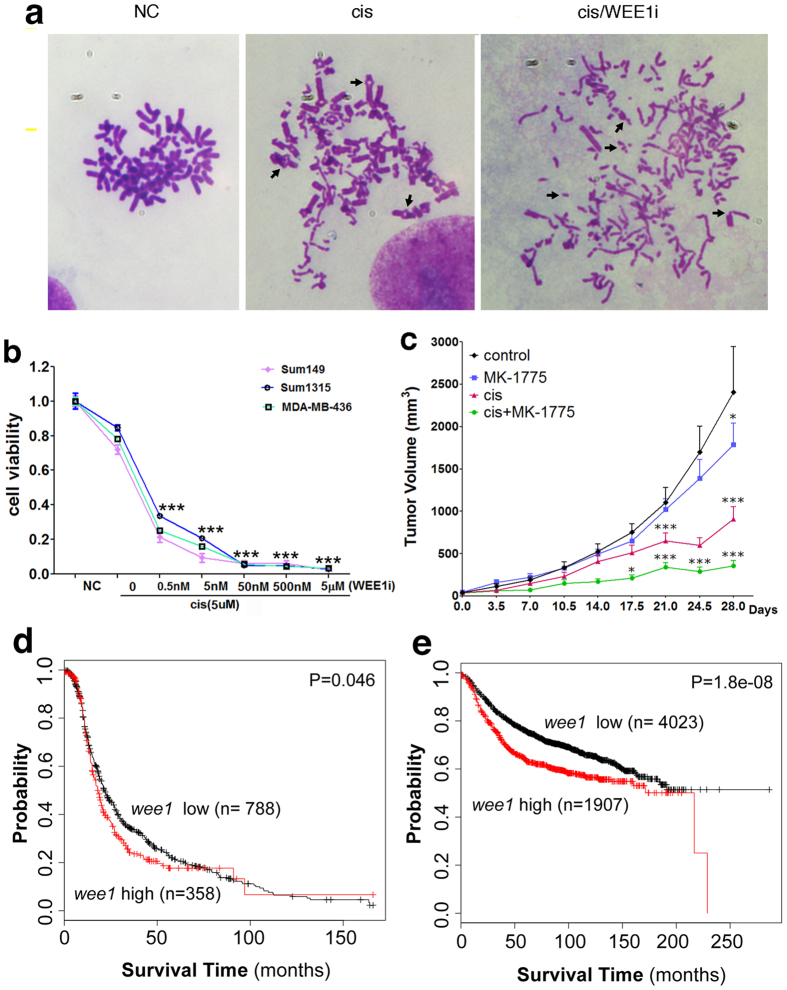
Antitumor efficacy of cisplatin and MK-1775 in human breast cancer cell lines and in xenograft model. (**a**) Chromosome spreads prepared from MDA-MB-231 cells that were mock, mono-cisplatin, and cisplatin/WEE1i double treated for 6 hours. Chromosomal abnormalities were observed in 30 out of 70 (42.9%) cisplatin treated, and 19 out of 36 (52.8%) cisplatin/WEE1i double treated cells. Arrows point to chromosome loops, which may be caused by chromatin crosslinking or chromosome fusion. In the double treated cells, majority of these abnormalities were replaced by chromosome fragmentation. This type of lesion is much more severe and may not be repairable, leading to lethality of cells. (**b**) Viability of cell lines to cisplatin and cisplatin/WEE1i treatment. Cells were treated with cisplatin and increasing doses of MK-1775 for 72 hours and assessed for cell viability by MTT assay. Comparison was made between mono-treatment of cisplatin: 5 μM, which results in about 80% viability) and cis/WEE1i. ***p < 0.001. (**c**) Nude mice bearing MDA-MB-231 xenograft tumors were treated with vehicle, MK-1775, cisplatin, or a combination of cisplatin and MK-1775 for 28 days and tumor volumes were measured. Data are presented as the mean of 8 tumors for each group ± SEM. (**d**,**e**) Kaplan-Meier relapse-free survival curve separates the tumors, i.e. ovarian cancer undergone therapy containing cisplatin (**d**) and breast cancer undergone chemotherapy (**e**), into two groups based on *wee1* expression. Data obtained from the Kaplan-Meier plotter database.

To evaluate the potential consequence of abrogation of cell cycle checkpoints for DNA replication initiation and G2 to M transition by cisplatin/WEE1i treatment, we conducted chromosome spread. The data indicated that 30 out of 70 (42.9%) cisplatin treated cells displayed multiple chromosomal abnormalities compared with untreated cells characterized by chromosome crosslinking, fusion, and aneuploidy (Fig. 7a). Of note, cells treated with cisplatin/WEE1i exhibited much more severe chromosome damage than cisplatin mono-treatment cells in that most chromosomes were already fragmented (Fig. 7a). The extensive chromosome damage is consistent with the more profound death after the cells were treated with cisplatin and WEE1i (Fig. 4d and Supplementary Fig. 5d). Similar profound effect of cisplatin/WEE1i treatment is also observed in other three TNBC cell lines: Sum149, Sum1315 and MDA-MB-436 (Fig. 7b), indicating that the cisplatin/WEE1i double treatment is applicable to more TNBC cell lines. We have also treated cells with cisplatin and shRNAs against *wee1*, and observed similar significant more lethality in the double treatment vs cisplatin mono-treated cells at most time points (Supplementary Fig. 7a–c), indicating the reduced viability caused by WEE1i is not due to off-target effects of the inhibitor. Next, we determined the efficacy of cisplatin, WEE1i, or combination of the two in xenograft model generated by implanting MDA-MB-231 cells into the mammary fat pad of nude mice. 4 weeks after the treatment with different drug combinations, the growth of tumors was evaluated by measuring tumor size over the course of treatment. The data indicated the combination of cisplatin and MK-1775 was substantially more effective at reducing tumor growth than either mono-treatment (Fig. 7c).

Consistent with its role of WEE1i in cell fate and cisplatin resistance probably, a higher levels of *wee1* correlate with a poor relapse-free survival stratified from a large public clinical microarray database of ovarian cancer patients undergone chemotherapy, which contained cisplatin, as well as breast tumor patients undergone chemotherapy (Fig. 7d,e)^36^,^37^. The data also shows that the survival outcomes are poorer in TNBC patients with higher levels of WEE1 (Supplementary Fig. 7d)^37^.

## Discussion

Cisplatin has been used for the treatment of many cancers, however, drug resistance frequently occurs through multiple mechanisms, which casts a major obstacle to its clinical application^7^,^11^,^12^. Thus, it is urgent to develope therapeutic approaches to overcome cisplatin resistance. It was shown that WEE1 kinase inhibition could overcome cisplatin resistance associated with high-risk TP53 mutations in head and neck cancer through mitotic arrest followed by senescence^38^. We also recently demonstrated that inhibition or knockdown of ATP7A, which is a copper transporting P-type ATPase, ATP7A could sensitize breast tumor cells to cisplatin^39^. We now show that kinases ATR, CHK1 and WEE1 when silenced confer sensitivity to cisplatin of multiple basal type breast cancer cells and cisplatin resistant MB-MDA-231 breast cancer cells. We provide the mechanism that the effect of cisplatin is first based on the critical effect of cisplatin on DNA replication: cisplatin targets DNA replication and causes replication stress. Then, this stress causes the activation of ATR, CHK1 and WEE1, which shuts down DNA replication origin firing and prevents cisplatin treated cells, although with massive DNA damage, from proliferation and therefore attenuate lethal effect of cisplatin.

The signaling pathway of ATR and CHK1 checkpoint kinases has a key role in suppressing replicative stress and DNA replication and can be targeted by several specific inhibitors^20^,^21^,^40^. WEE1 has its major role in G2-M transition traditionally^40^,^41^,^42^, but has an emerging role in control of DNA replication in the S phase^20^,^33^,^40^,^43^. The fact that ATR, CHK1 and WEE1 exert their function in DNA replication in common and they all showed up as top hits from our screen prompting us to further investigate our hypothesis about cisplatin and DNA replication. Here, we combined immunostaining of phosphorylated H2AX, chromatin-loaded RPA, staining of EdU and detection of ATR-CHK1 signaling as read out^20^ for cisplatin effect and DNA replication stress, and provided evidence that cisplatin treatment, in the early stage (4 h), causes mainly DNA replication stress, and proceeds to DSBs thereafter. DNA replication stress is characterized as inefficient DNA replication, which causes DNA damage and genome instability^20^,^44^.

A notable finding is that in the combination of cisplatin, WEE1 inhibition is more potent than the inhibition of ATR or CHK1 in killing TNBC cells. It has been shown that WEE1-deficiency could cause premature mitotic entry of cells^33^,^34^,^35^,^43^. Our data indicated that this remains the case for cells that are arrested in the S-phase by cisplatin treatment while such an effect is not observed upon the inhibition of ATR or CHK1. This data may count for the reason why WEE1 inhibition is more potent than inhibition of ATR or CHK1. Our further investigation indicated that the combination of cisplatin and WEE1 inhibition could also re-sensitize cisplatin-resistant to cisplatin. In this regards, our meta-analysis from a public clinical microarray database of both ovarian cancer patients and breast cancer patients undergone chemotherapy, which contained cisplatin^36^,^37^, revealed that lower levels of WEE1 correlates with longer relapse free period of time. This is consistent with our finding that knockdown of WEE1 has better therapeutic effect with cisplatin.

Recently, emerging lines of evidence suggested epithelial-mesenchymal transition (EMT) and the resulted cells with cancer stem cells (CSC) properties are responsible for chemo-resistance, metastasis and recurrence after therapy^45^,^46^,^47^,^48^,^49^. CSCs have been identified in a spectrum of tumors, including brain, skin, and intestinal tumors. It has also been suggested that tumor growth and metastasis in breast cancer can be attributed to these CSCs^50^,^51^. WEE1 has been suggested play a role to maintain a stem-like state and therapy-resistance^52^,^53^. Of note, a recent study has suggested EZH2, which belongs to the polycomb repressive complex 2 (PRC2) of Pc-G, as a therapeutic target of WEE1 inhibitor combinations in triple-negative and basal-like breast cancers, because EZH2 high expression in those cancers may provide a permissive environment for unscheduled mitosis, leading to mitotic slippage, apoptosis, and gross micronuclei formation^43^. Thus, WEE1 inhibition promotes both G1-S phase transition and mitotic entry of the cell cycle, which is related to potentially PRC1 and PRC2 of Pc-G, respectively.

In sum, our data showed that DNA damage induced by cisplatin treatment activates the DNA replication checkpoint mediated by ATR, CHK1 and WEE1, which arrests cells in the S phase and therefore prevents them from lethality caused by cisplatin. On the other hand, knockdown of ATR, CHK1 or WEE1 by siRNA or inhibition of them by using specific inhibitors causes re-firing of DNA replication and thus further enhance replicative stress and lead to more DNA damage, which eventually causes cancer cell death (Supplementary Fig. 8). The data also showed that although the combination of cisplatin with WEE1i, ATRi or CHK1i could overcome cisplatin resistance, cisplatin and WEE1i combination has better therapeutic effect than other two combinations. These data suggest that the levels of ATR, CHK1, especially WEE1, modulate cisplatin mediated cell death and survival, which is also reflected by our analysis of clinic data on ovarian cancer patients and breast cancer patients undergone chemotherapy, which contains cisplatin. Because cisplatin resistance frequently occurs^1^,^2^,^3^,^4^,^5^,^6^,^7^,^8^,^9^,^10^,^11^,^12^, our finding should have a significant impact in clinic applications in combating with cisplatin resistant cancers. As several other platinum-based drugs, such as carboplatin, are also used in treating various cancers, we will study their synergy with WEE1i in combating with breast cancer in near future.

## Methods

All methods were performed in accordance with the relevant guidelines and regulations by NIDDK, NIH, Bethesda, MD, USA, and the Faculty of Health Sciences, University of Macau, Macau SAR. China.

### Cell lines and cell culture

All human breast cancer cell lines (MDA-MB-231, SUM149, SUM1315, MDA-MB-436) were obtained from ATCC and cultured with DMEM (Life Technologies) supplemented with 10% FBS (Sigma) and 1% l-glutamine (Life Technologies). MCF10A immortalized mammary epithelial cells were obtained from ATCC and cultured with DMEM/F12 (1:1; Invitrogen) supplemented with 5% horse serum (Life Technologies), hydrocortisone (0.5 μg/mL; Sigma), EGF (20 ng/mL; Peprotech), insulin (10 μg/mL; Invitrogen), and cholera toxin (100 ng/mL; Sigma). The cisplatin-resistant breast cancer cell line MDA-MB-231 (Cis-R) was established by chronically exposing parental MDA-MB-231 cells to gradually increased concentrations of cisplatin (Sigma) starting from 0.1 μg/ml to 3 μg/ml for over one year until it became resistant.

### High-throughput screen

Two MDA-MB-231 cell lines, initially maintained independently in the National Cancer Institute and the Human Genome Research Institute, respectively, were used for this screen according a procedure described earlier^54^. Briefly, transfections were performed in 384 well plates (Corning 3570), and cell viability was measured using Cell Titer Glo (Promega). For transfections, 20 μL of serum free media containing Lipofectamine RNAi Max (0.07 μL) was added to wells containing siRNA (0.8 pmol). Lipid and siRNA were allowed to complex for 45 min at ambient temperature before addition of 700 cells in DMEM, 20% FBS to yield final transfection mixtures containing 20 nM siRNA in DMEM, 10% FBS.

The kinome screening campaign was conducted using the Ambion Silencer Select Human Kinase Library Version 4. This library targets 704 human genes with 3 siRNAs per gene^55^. Each siRNA is arrayed in an individual well. Cisplatin (10 μM, ~EC_30_, added in 10 μL of DMEM) or vehicle (10 μl DMEM) was added to the entire plate 24 h post-transfection and viability (Cell Titer Glo, Promega) was assayed 72 h later on a PerkinElmer Envision 2104 Multi-label plate reader. Ambion Silencer Select Negative Control #2 was incorporated on all screening plates for normalization (16 wells per plate; the median negative control value on each plate was used to normalize sample wells). Qiagen’s All Stars Cell Death control was incorporated as a positive transfection control (16 wells per plate). All screen plates exhibited assay z’-factors greater than 0.6.

To select candidate genes that modulate CPT activity, the log2 ratio of VO-treated cell viability (%siNeg) divided by CPT-treated cell viability (%siNeg) was calculated for each siRNA. Redundant siRNA Analysis (RSA) was then performed on the ratios to rank gene candidates in terms of their ability to sensitize MDA-MB-231 to CPT. A gene was considered a top candidate if its corresponding RSA p-value was <0.001 in either of the replicate screens.

Ingenuity Pathway Analysis (Ingenuity^®^ Systems, www.ingenuity.com) was performed to identify enriched pathways and protein-protein interactions among the top candidates. For IPA, a core analysis was performed using only direct relationships and used the 704 genes represented in the screen as background.

Knockdown was evaluated by TaqMan assay using a Quantstudio 12K Flex Real Time PCR system (Life Technologies). Each sample was evaluated in triplicate and the average Ct values were used to calculate knockdown compared to non-transfected cells. GAPDH was used for housekeeping. RNA was purified using an RNeasy Mini Kit (Qiagen) according to manufacturer instructions. cDNA was prepared using a High Capacity RNA to cDNA kit (Life Technologies). Taqman assays included HS00967506_m1 (CHEK1), HS00992123_m1 (ATR), HS011119384_g1 (WEE1), and 4352665 (GAPDH). qPCR was performed using Taqman Gene Expression Master Mix (Life Technologies).

### Lentivector-*wee1* short hairpin (sh)RNA transfection

MDA-MB-231 cells were cultured in 6-well plates for 24 hours to reach the confluence of ~70% when the transfection was performed. The sequences of *wee1* shRNAs are listed below. The *wee1* shRNA lentiviral vector pLKO.1 puro (Addgene) were prepared in accordance with the procedures described in the manufacturer’s instructions. Puromycin was used to select transfection positive 231 cells Then the selected cells were plated in 96-well plate for drug treatment or lysised for western-blot to check the WEE1 protein level. Lentiviral particles of empty vector were used as control.

**shRNA sequence**:

shRNA1 TTCTCATGTAGTTCGATATTT

shRNA2 TAATAGAACATCTCGACTTAT

### Chemicals and antibodies

The following chemical inhibitors were used at the indicated concentrations unless stated otherwise: WEE1 inhibitor MK-1775 (500 nM; Selleck Chemicals), CHK1 inhibitor SB 218078 (2 μM; Tocris), ATR inhibitor VE-821 (20 μM; Selleck Chemicals), CDK1 inhibitor RO-3306 (10 μM; Calbiochem), CDK inhibitor Roscovitine (20 μM; Selleck Chemicals), thymidine (2 mmol/L; Sigma-Aldrich), and nocodazole (100 ng/mL; Sigma-Aldrich), IdU (10 μg/ml, Sigma-Aldrich), CIdU (10 μg/ml, Sigma-Aldrich). The following antibodies were used: CHK1 (Santa Cruz Biotechnology), CHK1-pS345 (Cell Signaling), CHK2 (Santa Cruz Biotechnology), CHK2-pT68 (Cell Signaling), phospho-Histone-H2AX-Ser139 (γH2AX; Millpore), CDK1 (Santa Cruz Biotechnology), CDK1-pTyr15 (Cell Signaling), RPA70 (Abcam), phospho-(Ser) CDKs Substrate (pCDK Substrate; Cell Signaling), CDK2 (Santa Cruz Biotechnology), CDK2-pTyr15 (Abcam), RPA32-pT21 (Abcam), RPA32-pS33 (Abcam), RPA32-pS4/8 (Bethyl Laboratories), RPA32 (Bethyl Laboratories), WEE1 (Santa Cruz Biotechnology), α-Tubulin (Sigma-Aldrich), CIdU (rat anti-BrdU; Accurate Chemical), IdU (mouse anti-BrdU; Becton Dickinson).

### Cell viability assay

Cells were seeded at a density of 2–3 × 10^4^ cells per well in 24-well plates and incubated at 37 °C in humidified 5% CO2 for 24 hours. Then the indicated drugs were added to the cells for the designated time and concentration. Cell viability was assessed by methylthiazolydiphenyl-tetrazolium bromide (MTT) assay. A 0.5-mg/mL solution of Thiazolyl Blue (Sigma) in phenol-free DMEM was added to cells at 37 °C for 1 hour. The substrate was then dissolved in isopropanol and absorbance was measured with a spectrophotometer at 570 nm. All MTT assays were performed 3 times in triplicate.

Cell apoptosis after indicated drug treatment was detected by flow cytometry with Annexin V conjugates following manufacturer’s instructions (Life Technologies).

### Immunostaining and western blot

For immunostaining, cells growing on 12 mm coverslips were fixed in formaldehyde 4% (VWR) for 20 minutes at room temperature, washed, permeabilizedin 0.5% Triton X-100 in PBS for 10 minutes, washed, and blocked in IFF [1% bovine serum albumin, 2% FBS in PBS] followed by incubation with primary antibodies for 1 to 3 hours, and secondary fluorescently labeled antibodies (Alexa fluorophores, Life Technologies) for 1 hours at room temperature. PBS-Tcontaining 4′,6-Diamidino-2-Phenylindole Dihydrochloride (DAPI, 0.5 mg/ml) was applied for 5 min at room temperature to stain DNA. Coverslips were mounted on glass slides using ProLong^®^ Gold Antifade Mountant (Life Technologies). Images were acquired on a Leica DMR microscope. Wherever specified in Figure legends, pre-extraction was carried out. For pre-extraction, cells were washed once with PBS and incubated with ice-cold PBS containing Triton X-100 (0.2%) for 1 min on ice prior to fixation. When Click-it reactions were combined (EdU), these were performed prior to incubation with the primary antibodies following manufacturer’s instructions (Life Technologies). Fluorescent signal intensity and colocalization of signals were measured and analyzed using ImageJ.

Western blot analysis was performed with Licor (Lincoln, NE).

### Chromosome spread

For preparation of chromosome spreads, MDA-MB-231 cells were treated with 100 ng/ml colcemid solution for 2 hours before harvesting. Cells were treated with 0.56% KCl for 10 minutes at 37 °C, fixed in methanol: acetic acid (3:1), and dropped onto glass slides. Chromosomes were stained with Giemsa and morphology were assessed under a Leica microscope with a 100X objective.

### Xenograft experiments

MDA-MB-231 cells were injected into the fourth mammary fat pad of female nude mice (5 to 6 weeks) at 1 × 10^6^ cells/100 μl/spot. When the tumors became palpable, mice were individually identified and randomly assigned to treatment groups of 5 mice: 1) PBS (control); 2) MK-1775 (in 0.5% methylcellulose, 30 mg/kg p.o., twice a week); 3) cisplatin (in PBS, 6 mg/kg i.p., twice a week); or 4) a combination of cisplatin and MK-1775. Tumor volumes were measured twice a week with a caliper and calculated as previously described^56^. Tumor volume was calculated in mm^3^ by the following equation: V = (*a*^2^ * b)/2, where *a* is the width of the tumor (small diameter), and *b* the length (large diameter), both in millimeters. The protocols for animal studies were approved by the Animal Care and Use Committee (ACUC) of the NIDDK and University of Macau, respectively.

### Statistical analysis

All statistical tests were conducted with GraphPad Prism version 5.0. Statistical significance was determined using the Student’s T-test for experiments comparing two groups. Comparisons among groups were analyzed using analysis of variance (ANOVA). Comparisons between different groups were analyzed using 2- way ANOVA. Unless stated otherwise, *P* values were 2-tailed and considered significant if *P* < 0.05. Error bars represent SEM of 3 experiments unless stated otherwise.

## Additional Information

**How to cite this article:** Zheng, H. *et al*. WEE1 inhibition targets cell cycle checkpoints for triple negative breast cancers to overcome cisplatin resistance. *Sci. Rep.* **7**, 43517; doi: 10.1038/srep43517 (2017).

**Publisher's note:** Springer Nature remains neutral with regard to jurisdictional claims in published maps and institutional affiliations.

## Supplementary Material

Supplementary Information
