# Supplementary material for: WEE1 inhibition targets cell cycle checkpoints for triple negative breast cancers to overcome cisplatin resistance
Source: Sci Rep. 2017 Mar 6;7:43517. doi: 10.1038/srep43517 (PMC5338009; doi:10.1038/srep43517)
Supplement: Supplementary Information [file srep43517-s1.pdf]

WEE1 inhibition targets cell cycle checkpoints for triple negative breast cancers to overcome cisplatin resistance

Hongping Zheng[2], Fangyuan Shao[1], Scots Martin[3], Xiaoling Xu[1], and Chu-Xia Deng[1,2]

1. Faculty of Health Sciences, University of Macau, Macau SAR, China.
2. Genetics of Development and Disease Branch, National Institute of Diabetes and Digestive and Kidney Diseases, National Institutes of Health, USA.
3. Division of Pre-Clinical Innovation, National Center for Advancing Translational Sciences (NCATS), National Institutes of Health, USA

Correspondence:

Chu-Xia Deng

Faculty of Health Sciences, University of Macau, Macau SAR, China.

cxdeng@umac.mo

Supplementary Figures and legends

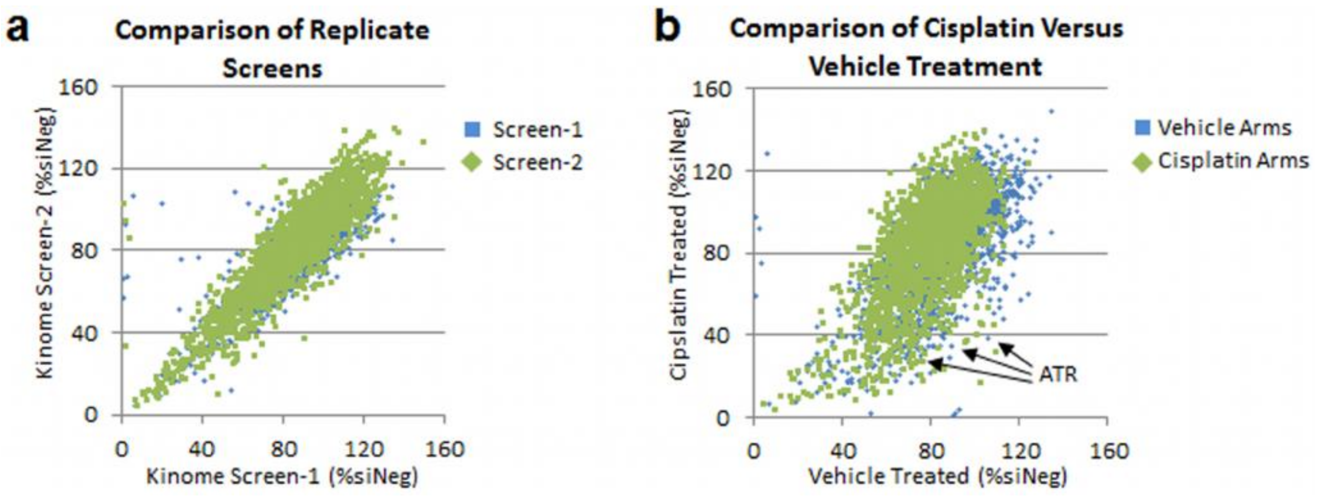

Supplementary Figure 1. Evaluation of replicate kinome siRNA screens. (**a**) Replicate screens correlate well (vehicle arm R=0.83; cisplatin arm R=0.90). (**b**) A comparison of treated vs non-treated screen arms revealed a number of siRNAs that appear to enhance cisplatin toxicity, including ATR.

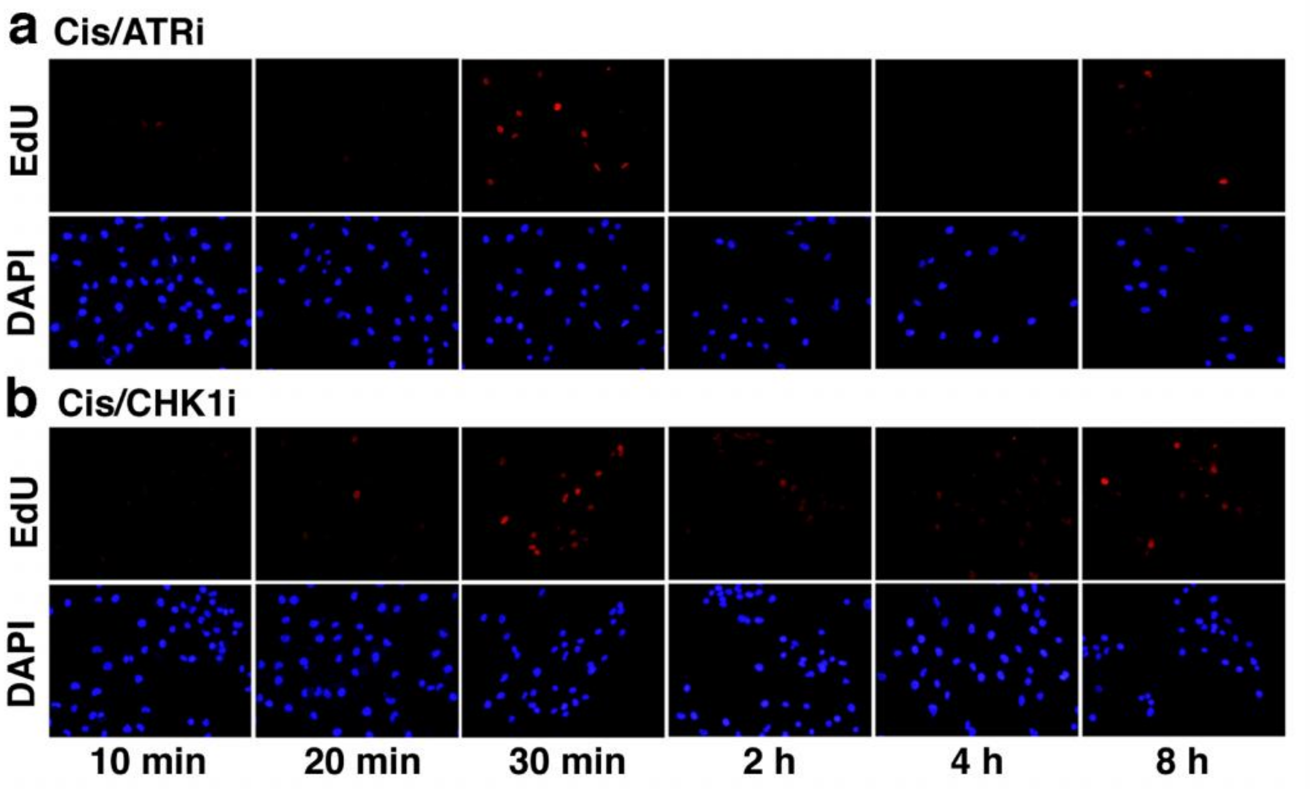

Supplementary Figure 2. Inhibition of WEE1 activity restore the fork activity more effectively than ATR/CHK1 inhibition in cisplatin treated cells. (**a, b**). MDA-MB-231 cells were incubated with cisplatin (5 μg/ml) for 3 hours followed by incubation with fresh medium with ATR (**a**) or CHK1 (**b**) inhibitor for indicated times and EdU incorporation for additional 10 min. Cells were fixed for subsequent staining. Nuclear DNA was counterstained by DAPI, EdU was detected through the Click-iT reaction. EdU intensity per nucleus were obtained.

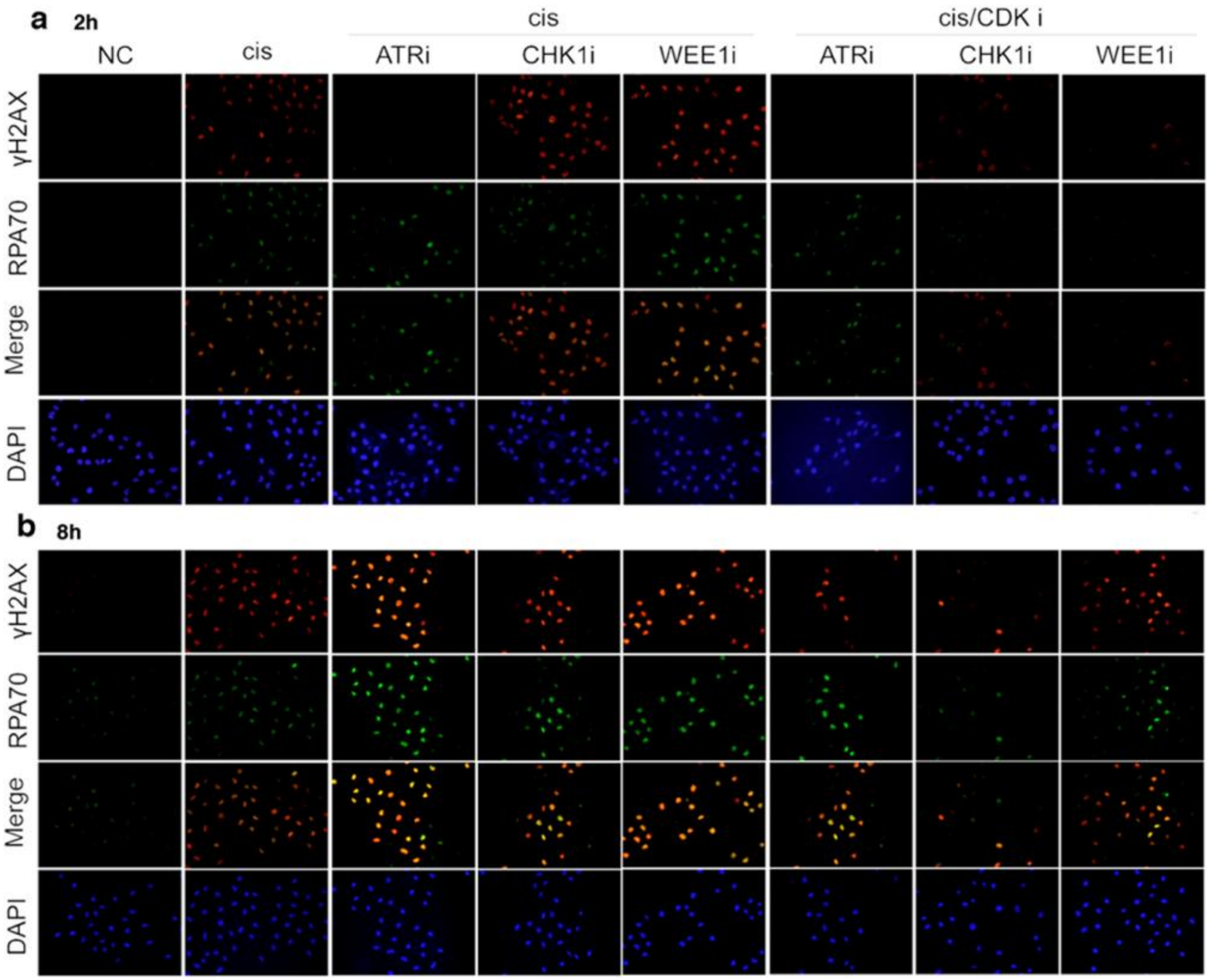

Supplementary Figure 3. Inhibition of ATR/CHK1/WEE1 activity can cause more DNA damage in cisplatin treated cells. (**a**, **b**). MDA-MB-231 Cells were treated with indicated drug(s) (top) for 2 (**a**) or 8 (**b**) hours, and immunostained with γH2AX and RPA70 antibodies after pre-extraction. Nuclear DNA was counterstained by DAPI.

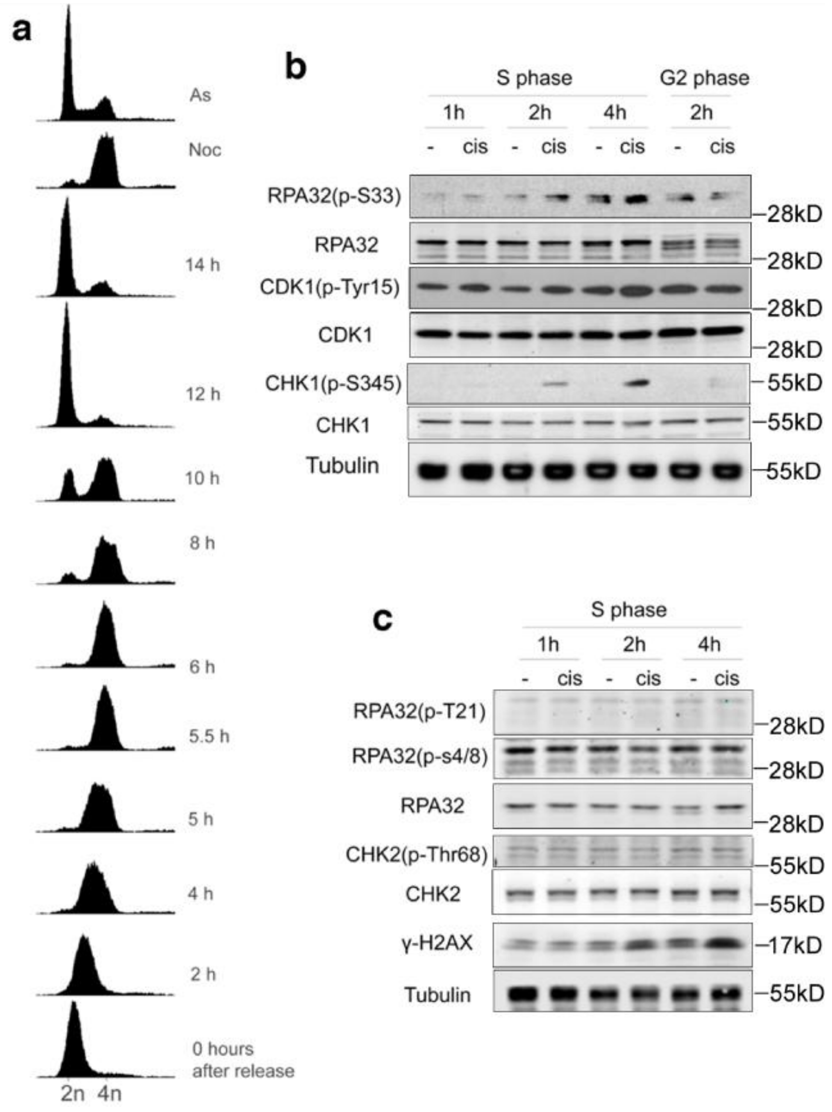

Supplementary Figure 4. (**a**). Cell-cycle distribution analysis of propidiumiodide-stained cells by flow cytometry. As, asynchronous cells; Noc, nocodazole-treated cells. (**b**, **c**). Western blot analysis of various proteins in cells at S phase or G2 phase in the presence or absence of cisplatin. The cells were synchronized and released and treated as indicated and collected for western blot using indicated antibodies. 0 hour to 5 hours after release is considered as S phase; 5.5 h to 8 h is considered as G2 phase.

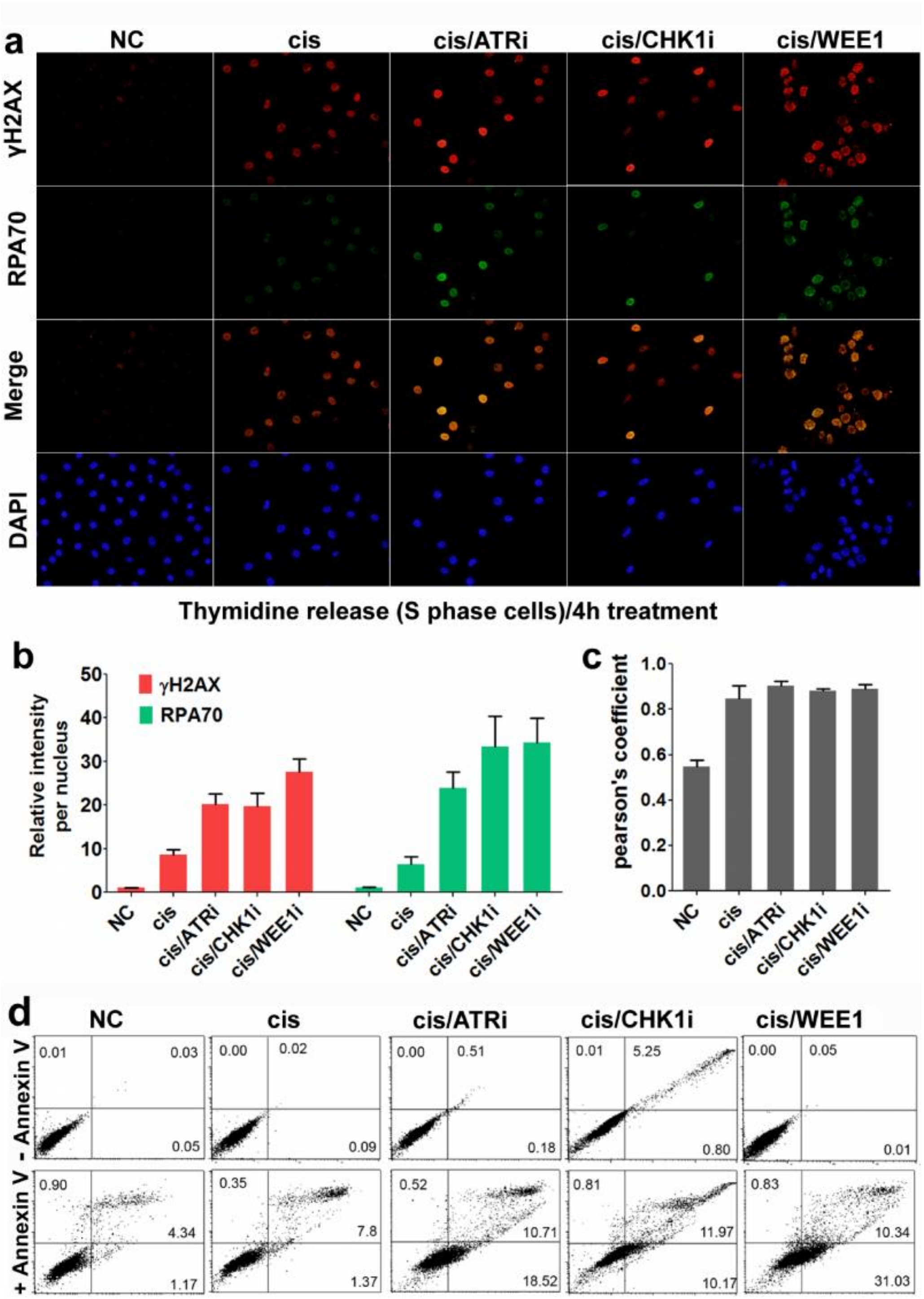

Supplementary Figure 5. Inhibition of ATR/CHK1/WEE1 activity can cause more DNA damage in cisplatin treated S-phase cells. (**a**). MDA-MB-231 cells were synchronized by a double thymidine block after which indicated drug(s) (top) was added for an additional 4 hours

and then cells were immunostained with γH2AX and RPA70 antibodies after pre-extraction. Nuclear DNA was counterstained by DAPI. (**b**). Quantification of average γH2AX or RPA70 values relative to untreated cells of three separate experiments as in A represented as the mean ± SEM. (**c**). Pearson's coefficient is shown as the quantification of γH2AX and RPA70 co-localization of three separate experiments as in (**a**) represented as the mean ± SEM. (**d**). MDA-MB-231 cells were treated with the indicated drugs for 24 hours and harvested for flow cytometric detection of apoptosis with Annexin V conjugates**.**

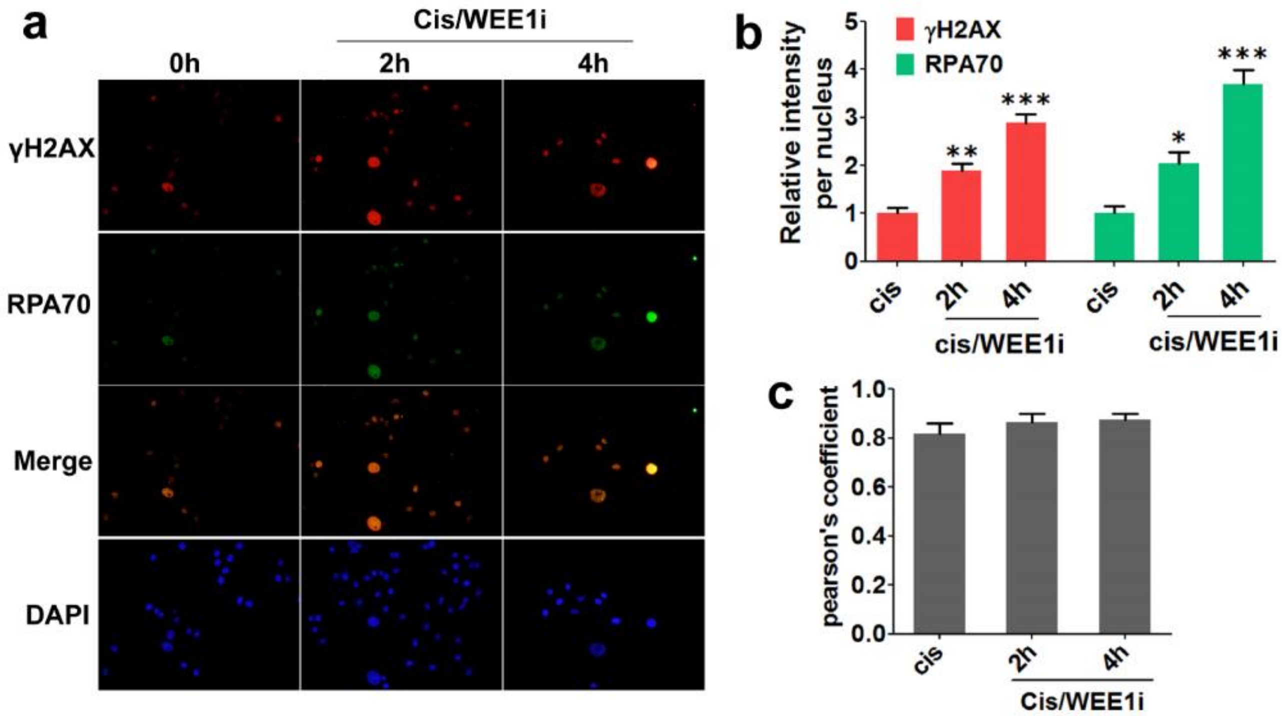

Supplementary Figure 6. Response of cisplatin-resistant cells to WEE1 inhibition. (**a**) Cisplatin-resistant 231cis-R(2) cells were cultured in medium with cisplatin. The cells were additionally treated with WEE1 inhibitor for indicated times, and then cells were immunostained with γH2AX and RPA70 antibodies after pre-extraction. Nuclear DNA was counterstained by DAPI. (**b**). Quantification of average γH2AX or RPA70 values relative to cells incubated only with cisplatin of three separate experiments as in (**a**) represented as the mean ± SEM . (**c**). Pearson's coefficient is shown as the quantification of γH2AX and RPA70 colocalization of three separate experiments as in (**a**) represented as the mean ± SEM.

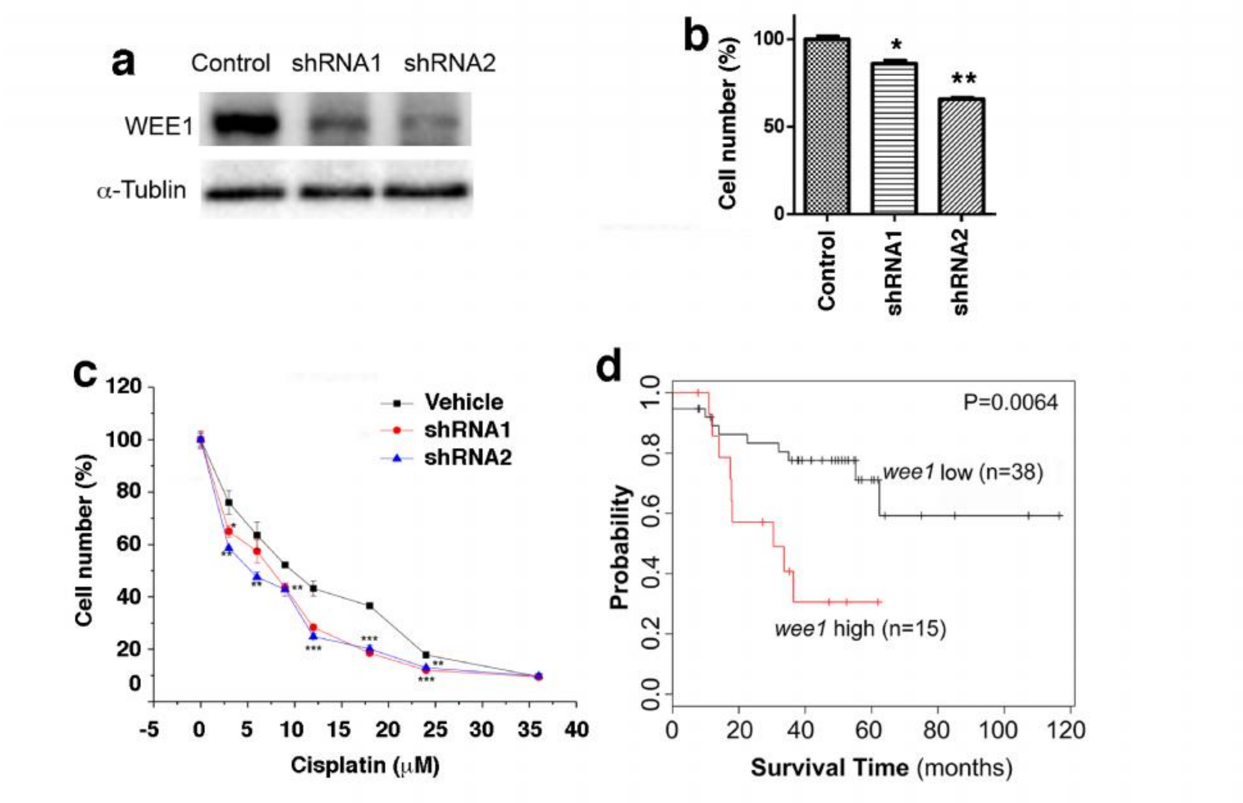

Supplementary Figure 7. WEE1 silencing significant increased the sensitivity of MDA-MB-231 cells to cisplatin. (**a**). Western blot confirmed reduced protein levels of WEE1 by lentiviral-*wee1* shRNAs. (**b**). MDA-MB-231 cells were cultured and transfected by lentiviral-*wee1* shRNAs, and then 3 days later cells were assessed for cell viability by the MTT assay. Data represent mean ± SEM normalized to cell viability in control cells which were transfected with lentiviral particles of empty vector. (**c**). MDA-MB-231 cells were cultured and transfected by lentiviral-*wee1* shRNAs, treated with cisplatin by indicated concentrations, and 3 days later cells were assessed for cell viability by the MTT assay. Data represent mean ± SEM normalized to cell viability in control cells which were transfected with lentiviral particles of empty vector. * p<0.05, ** p<0.01, *** p<0.001 for student t-test. **(d)** Kaplan-Meier relapse-free survival curve separates TNBCs based on their WEE1 expression levels. Data obtained from the Kaplan-Meier plotter database [37].

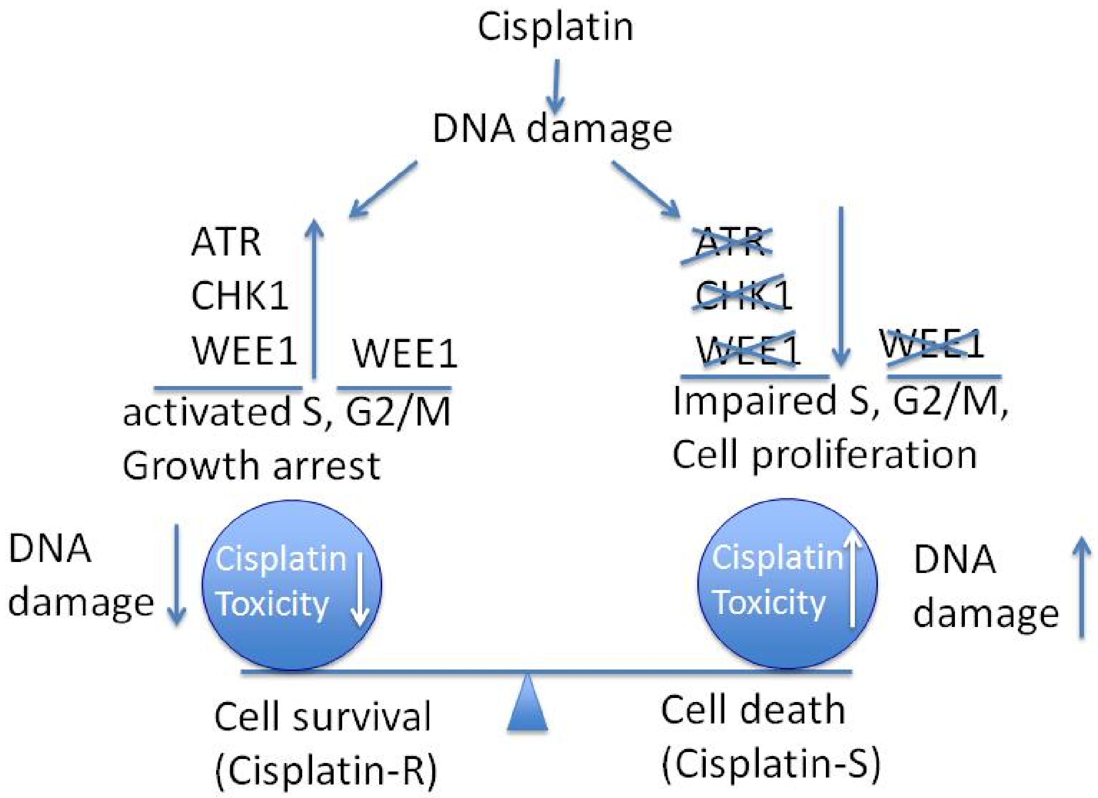

Supplementary Figure 8. A model summarizing action of cisplatin in inducing DNA damage, cell cycle arrest upon the activation of DNA replication checkpoint. Inhibition of ATR, CHK1 or WEE1, allows cell proliferation with extensive more DNA damage, leading to cell death.
